# Immunological and anti-oxidant profiles of malarial children in Abuja, Nigeria

**DOI:** 10.37796/2211-8039.1010

**Published:** 2021-03-01

**Authors:** Idris Nasir Abdullahi, Sanusi Musa, Anthony Uchenna Emeribe, Musa Muhammed, Jelili Olaide Mustapha, Halima Ali Shuwa, Shamsuddeen Haruna, Sharafudeen Dahiru Abubakar, Olusoji Matthew Adeyemi Billyrose, Mustapha Bakare

**Affiliations:** a Department of Medical Laboratory Science, Faculty of Allied Health Sciences, Ahmadu Bello University, Zaria, Nigeria; b Department of Medical Laboratory Science, Faculty of Allied Medical Sciences, University of Calabar, Calabar, Nigeria; c Department of Medicine (Immunology Unit), Ahmadu Bello University, Zaria, Nigeria; d Biological Sciences Department, Faculty of Science, University of Alberta, Edmonton, Canada; e Department of Community Health, Federal University, Dutse, Nigeria; f Department of Medical Laboratory Services, University of Abuja Teaching Hospital, Abuja, Nigeria

**Keywords:** malaria immunity, cellular immunity, oxidative stress, sub-Saharan Africa

## Abstract

**Background:**

The clinical symptoms, cellular immune response, and serum cytokine homeostasis during falciparum malaria among children living in endemic regions depend on the parasite densities. This study aims to evaluate the CD4^+^ and CD8^+^ T cells, leucocytes subpopulations, IL-6, IL-10 and biomarkers of oxidative stress among children infected with varying grades of malaria attending the University of Abuja Teaching Hospital and National Hospital, Abuja, Nigeria.

**Materials and methods:**

This case-control study involved blood samples collected from 165 children (between 5 and 12 years). This comprised 45 children with mild malaria, 40 each with moderate, severe malaria and apparently healthy (control). Serum cytokines, ferritin, malonaldehyde (MDA), ascorbate, α-tocopherol levels were determined by Enzyme-Linked ImmunoSorbent Assay (ELISA). Leucocytes differentials and CD4^+^/CD8^+^ T cells counts were enumerated by automated hematology analyzer and flow cytometry, respectively.

**Results:**

All malarial children had only *Plasmodium falciparum*. The male to female ratio of children with mild malaria was 1.5:1 (mean ± SD age of 8.5 ± 1.9 years). However, other groups had 1:1 male to female ratio and mean ages of 9.2 ± 2.3, 9.8 ± 2.2, 8.5 ± 1.5 for children with moderate, severe malaria and control, respectively. There was a positive but not significant association of neutrophils and monocytes with the 3 grades of malaria parasitemia (p>0.05). There was a negative and significant correlation between severe malaria and lymphocyte count (p = 0.048; r = −0.647). However, there was positive and significant correlation between eosinophil with moderate (p = 0.03; r = 0.994) and severe malaria (p = 0.006; r = 0.825). There was a significant decline in serum ascorbate with increased malaria density (p<0.0001). However, there was no difference in the serum α-tocopherol concentration within the 4 groups of children (p = 0.182). Serum ferritin and MDA significantly elevated with an increase in malaria density (p<0.0001). There was a significant decline in CD4^+^ T and CD8^+^ T cells counts with an increase in malaria densities (p<0.0001). Serum IL-10 and IL-6 significantly elevated with increased malaria density (p<0.0001).

**Conclusion:**

Based on these findings, severe malaria was significantly associated with declined CD4^+^ and CD8^+^ T cell counts, upregulation of IL-6, and high serum levels of oxidative stress biomarkers.

## 1. Introduction

The World Health Organization (WHO) affirms that malaria constitutes a global health threat with 228 million cases and 405,000 deaths in 2018 [1]. The African sub-region accounted for approximately 93% of the global burden of malaria and 67% of malaria mortality occurred in children under five years [1–3]. It was reported that 6 African countries accounted for more than 50% of all global malaria cases, with Nigeria (25%) recording the highest cases [1]. In addition, Nigeria accounted for about 24% of all malariarelated mortality [1]. In Nigeria, the rise in the persistence of malaria endemicity has been linked to the poverty and well-being of the populace [3]. Complications such as hypoglycemia, impaired consciousness, anemia, and respiratory distress increase the risk of mortality in children afflicted with severe malaria. Because of the disproportionate distribution of malaria, it is the most important parasitic infection that attracts the greatest global attention [4]. Of the six malaria-causing species of the protozoa - Plasmodium, *Plasmodium falciparum (P. falciparum)* accounted for most morbidities and consequently, highest mortality rates in Africa and other high-risk regions of the world [5,6]. Therefore, infection with P. *falciparum* requires quick recognition, proper treatment and effective patient management [7].

Malarial infection can potentially lead to death if not treated promptly due to its progression into severe malaria anemia (SMA) [8]. In SMA, there is impaired immunity which results in lysis of both infected and uninfected erythrocytes, thereby reducing the hemoglobin level of the host by 20–50% [8]. In this instance, the management of this form of malaria-induced morbidity is mainly by blood transfusion. Furthermore, the reduction of hemoglobin levels has not been identified as a factor in the production of defective red blood cells. However, studies have shown that SMA is associated with parasite density in the organs and not the assumed number of blood-borne parasites in circulation [9].

Cellular and antibody-mediated immunity, innate, and adaptive cells play a crucial role in the immune responses to the stages of the malaria parasite life cycle and the development of immunopathogenesis of malaria [6,10]. Immune response to the parasite is associated with an individual’s age, as well as the degree of exposure to malaria, which makes people living in malaria-endemic regions resistant to symptomatic malaria, thereby developing asymptomatic infections afterward [10].

In malarial infection, innate and acquired immune responses are stage-specific and complex; the preerythrocytic (skin and liver) responses against sporozoites is distinct, while the erythrocytic responses against the merozoites are clearly different [6,10]. Through modulation of immune responses, *P. falciparum* interferes with maturation and proliferation of antigen-presenting cells (APCs) in addition to the impairment of dendritic cell activity, thereby resulting in the proliferation of “tolerant” T-cell phenotype [10]. Through the interference of the activation of liver-stage immunity, uptake of infected erythrocytes by dendritic cells weakens immune response during blood-stage malaria [10].

Oxidative stress in malaria can be attributed to two main mechanisms, viz; increased number of parasites in the erythrocyte as a result of high metabolic rate, and the host’s potential immune response to the infection [11]. There is a release of biomarkers of oxidative stress such as reactive oxygen species (ROS) and reactive nitrogen species (RNS) via host-parasite interaction. This ultimately stimulates the immune system with the possibility of causing red cell destruction through the induction of oxidative damages [12]. Therefore, red blood cells infected with plasmodium parasite are under constant oxidative stress utilized by the parasite with a resultant rise in antioxidant levels in the blood [12].

While some studies suggested that oxidative stress during malaria plays a protective role, others opposed that it plays a deleterious role by worsening the pathological outcome of the infection [12]. In malaria, oxidative stressors levels such as malondialdehyde (MDA) and superoxide (SOD) are higher while catalase levels are lower [11,12]. Due to the production and accumulation of peroxide (H_2_O_2_) through the activity of SOD and catalase, capable of reacting with several molecules to form free radicals, there is a need to remove these radicals using substances with anti-oxidant properties such as the ascorbic acid. In inflammation, leukocytes, alpha-1-acid glycoproteins, IL-6, and Vitamin C have similar concentrations [13]. Vitamin E is activated by Vitamin C which mops-up the products of oxidative stress/free radicals because their persistence could facilitate to severe malaria [14]. Therefore, clues of pathophysiological processes in malarial individuals could be extrapolated from their antioxidant status [14]. Aside from this, vitamin E and dietary carotenoids have been shown to be strong immune modulators in humans.

In children, previous studies have reported varying findings on the roles of pro- and anti-inflammatory cytokines in the pathogenesis of malaria and disease outcomes [15–17]. However, severe malaria has repeatedly been reported to cause a decline in CD4^+^ T cell counts and induction of anemia through CD8^+^ T cell-dependent plasmodial clearance and red cells removal in the Spleen [8,17]. These invariably lead to a rise in oxidative stress biomarkers [12,17]. Hence, it is hypothesized that an increase in malaria severity or density is associated with the upregulation of oxidation stress, proinflammation and downregulation of cellular immune function.

Given that a very larger proportion of the Nigerian population are at high risk of contracting malaria and children are most susceptible to malaria severity, this study would be relevant and generate interest in this setting, region and Sub-Saharan Africa. Specifically, there is a paucity of data that demonstrates the interplay of malaria densities with cellular immunity, cytokines and oxidative stress among Nigeria children. Hence, this work was instigated with the aim to determine the CD4^+^ and CD8^+^ T cells, leucocytes subpopulations, IL-6, IL-10 and biomarkers of oxidative stress among children infected with varying grades of malaria attending the University of Abuja Teaching Hospital and National Hospital, Abuja, Nigeria.

## 2. Materials and methods

### 2.1. Study area

This study was conducted during a 4-month period - 28th February to 30^th^ June 2019 at the University of Abuja Teaching Hospital (UATH), Gwagwalada and National Hospital Abuja (NHA), Nigeria. The UATH and NHA have 500- and 850-bed capacities, respectively. The UATH is located at 8.9531° N, 7.0614° E whileNHAis located at 9.0388° N, 7.4631° E. These are the two prominent tertiary hospitals situated at Nigeria’s Federal Capital Territory. The climate of Abuja is quite reminiscent of other tropical areas with 2 distinct seasons - the wet and dry season. The temperature ranges from 30 °C to 37 °C during the day and 18 °C–25 °C at night, with the highest temperature usually experienced in the month of March. The mean total rainfall is approximately 1650 mm/annum with more than 60% of this rain falling between July to September [18].

### 2.2. Study design

This was a hospital-based case-control study.

### 2.3. Sample size calculation

The minimum sample size for this study was determined from a previous case-control study by Efunshile *et al* [19] which reported 2.2% malaria among 6–10 years children (case) and 23.2% among <5 years children control. The calculated size at 80% power and 95% confidence interval was 39 each. However, 125 malarial children and 40 controls were recruited.

### 2.4. Study population

Participants were enrolled using stratified randomization. Stratified randomization strata were constructed based on values of malaria density and a randomization scheme was performed separately within each of the 4 strata to avoid imbalance in participants’ selection. They comprised of one hundred and sixty-five children within 5–12 years categorized into four groups, viz;

- Forty-five children with laboratory-confirmed with mild Plasmodium falciparum malaria.
- Forty children with laboratory-confirmed with moderate Plasmodium falciparum malaria.
- Forty children with laboratory-confirmed with severe Plasmodium falciparum malaria.
- Forty children without malaria history for the past 1 year. This was based on routine hospital check-up records, as well as information from parents attesting that children were neither sick nor on any treatment within the past 12 months. These children were entirely from the affluent who reside in the mainstream city of the federal capital (Abuja) of Nigeria.

All participants were identified and selected by nurses, pediatricians, and family physicians of these hospitals.

### 2.5. Clinical and laboratory classification criteria

#### 2.5.1. Mild malaria

Uncomplicated with mild fever.

Malaria density count <10,000 parasites/μL.

No anemia.

No abnormal blood chemistry.

No urinary proteinemia.

#### 2.5.2. Moderate malaria

High body temperature (39 °C) for ≥3 days.

Mild anemia (9–11 mg/dL).

No abnormal blood chemistry.

No urinary proteinemia.

Malaria density count between 10,000 parasites/μL and 100,000 parasites/μL.

#### 2.5.3. Severe malaria

Proteinuria (+).

Severe anemia (8 mg/dL).

Hyperbilirubinemia (Bilirubin >3 mg/dL).

High serum potassium (5.5 mmol/L).

Malaria density count of >100,000 parasites/μL.

### 2.6. Selection criteria of participants

Enrolment was based on participants who had no prior history of any immunodeficiency infections or diseases such as HIV/AIDS, diabetes mellitus, tuberculosis, typhoid, HCV and HBV. All participants were not on anti-malarial chemotherapy prior to sampling.

### 2.7. Ethical approval and consent

Ethical approval for this study as mandated was obtained from 2 sources; the Human Ethical Research Committees of UATH (approval number: FCT/UATH/HREC/PR/613) and NHA (approval number: NHA/ADMIN/259/III.VII), while written consent was provided to individual parents/guardians of children. Participants’ confidentiality and privacy were entirely protected, no third party had access to participants’ data. All data were analyzed anonymously and saved in a personalized google cloud account. After analysis, all data were permanently deleted from the internet and computers.

### 2.8. Data collection and cleaning

Age and sex of children were collected from their parents/guardians through a structured questionnaire. Whereas body mass indices (i.e. weight/squared height) were assessed by nurses. Data cleaning was done to identify and correct errors during collection to minimize their effects on the results. We screened and edited abnormal or faulty data such as outliers and were corrected by logarithmic transformation.

### 2.9. Laboratory analytical protocols

#### 2.9.1. Sample collection and preparation

Five milliliters (5 mL) blood samples were collected from all children. Out of this, 2 mL was dispensed into an Ethylene Diamine Tetraacetic Acid (EDTA) anticoagulant container which was used for CD4^+^ T cell counts and complete blood counts and differentials. The remaining was dispensed into plain container (with no anticoagulant). Of which, serum was harvested by centrifugation at 10000 *g* for 10 min. Thereafter, it was used for cytokine and oxidation biomarkers measurement. Samples were analyzed within 1 h of collection.

#### 2.9.2. Malaria detection and speciation

Malaria parasites were detected and identified microscopically using Giemsa stained thick and thin blood films, respectively, as described by Cheesbrough [20].

Thick films were used to detect malaria parasites in the blood samples. Three drops of blood were placed in the center of a clean grease-free microscope slide to cover an area of 15 mm in diameter. The smears were allowed to air-dry and then flooded with 10% Giemsa stain which was retained for 10 min before the stains were washed off with water. Parasite species and morphology were determined by microscopic examination of thin films which were also prepared with the 10% Giemsa stain. Microscopic examinations of the stained slides were done using an oil immersion objective lens (×100 magnification).

The parasite density in all samples was determined as previously described by Cheesbrough [20]. Certified microscopists with over 5 years of working experience in malaria microscopy did multiple-blinded readings for validation of the presence or absence of parasites and estimated the parasite densities. Positive control slides were included in all batches of microscopy.

Parasite densities (parasite/μL of whole blood) were then calculated as follows:

- The number of malaria counted/WBC counted × WBC count μL of the participant.
- Furthermore, parasite densities for all participants were calculated using assumed WBC counts of 8.0 × 10^9^/L of blood; all set by WHO to be used conveniently in facilities that lack the tools to determine patients’ absolute full blood count value [21].
- The number of parasites seen per field were counted and the density scored as <10,000 parasites/μL (mild); between 10,000 parasites/μL and 100,000 parasites/μL of whole blood (moderate) and >100,000 parasites/μL (severe) [21].

#### 2.9.3. Estimation of Il-6 and IL-10 by enzyme-linked immunosorbent assay

Serum IL-6 and IL-10 concentrations were measured by sandwich ELISA based on the protocol described by kit manufacturer (RayBio Human ELIS kit ®, Norcross, GA, USA). Accordingly, IL-10 (Catalog code: ELH-IL10) and IL-6 (Catalog code: ELH-IL6) were investigated.

#### 2.9.4. Flow cytometry for CD4^+^ and CD8^+^ T cells

To generate absolute counts of CD4^+^ and CD8^+^ T cell lymphocytes, a dual-platform method (using an automated hematological analyzer and flow cytometer) was utilized. A three-color flow cytometric analysis panel was done using a BD FACS™ brand flow cytometer (Franklin Lakes, USA). Before data acquisition, instrument parameters were checked and optimized using CaliBRITE beads (Becton Dickinson).

#### 2.9.5. Determination of serum ascorbate, malondialdehyde, α-tocopherol and ferritin concentration

Serum Ascorbic acid (Catalogue code: 1520-ASC-1; Alpha Diagnostics Inc, San Antonio, USA), Malondialdehyde (Catalogue code: EU2577; ElisaGenie ®, London UK) and α-Tocopherol (Catalogue code: UNEB0052; ElisaGenie ®, London UK) levels were estimated using competitive ELISA kits. While serum ferritin levels were measured using a sandwich ELISA kit (Catalogue code: 1810, Alpha Diagnostics Inc. San Antonio, USA). These analytes were assayed based on kits manufacturer’s instructions.

#### 2.9.6. Leucocyte counts and differentials

Full blood counts and leucocyte differentials were analyzed using Mindray BC-5000 (Shenzhen, China) automated hematology analyzer following the manufacturer’s operational guidelines. All samples were analyzed within 30 min of collection. Daily quality control for all laboratory procedures such as flow cytometry and ELISA including blood smear microscopy staining and automated hematology analyses daily quality control ensured.

### 2.10. Statistical analysis

All data were inputted into Microsoft Excel then exported to Medcalc software V.19.0.7 to analyze continuous variables as mean ± SD. We employed One-way ANOVA to define the difference between mean ± SD across study groups. Pearson’s correlation test was used to determine the association between leucocytes differentials and the various malaria densities. Dunnett’s Multiple Posttest was used to determine where differences existed. *P* values < 0.05 were considered statistically significant at a confidence interval of 95%.

## 3. Results

### 3.1. Characteristics of study participants

Table 1 shows the characteristics of the study population. There was a total of 165 participants <12 years (range 5–12years) which comprised of four groups; viz, 45 children with mild malaria, 40 with moderate and 40 with severe malaria and 40 age- and gender-matched apparently healthy children which served as control. The male to female ratio of children with mild malaria was 1.5:1 (mean ± SD = 8.5 ± 1.9 years), whereas, other groups had 1:1 male to female ratio and mean ages of 9.2 ± 2.3 years, 9.8 ± 2.2 years, 8.5 ± 1.5 years for children with moderate, severe malaria and control, respectively. (Table 1). The mean Body Mass Index (BMI) of participants with mild malaria was 19.6 ± 3.5 kg/mm^2^, then 18.8 ± 3.3 kg/mm^2^, 19.9 ± 2.1 kg/mm^2^, and 19.3 ± 4.3 kg/mm^2^ for those with moderate, severe and the controls, respectively (Table 1). Using one-way ANOVA, the mean age of the study groups significantly varied across the study groups (p = 0.008). However, there was no difference in BMI and all groups (p = 0.513). The mean malaria parasite densities of participants with mild malaria were 2754 ± 125 parasites/μL, then 16,254 ± 2520 parasites/μL and 227,651 ± 14,533 parasites/μL for those with moderate and severe malaria, respectively.

### 3.2. Leucocyte differentials of study participants based on malaria severity

Table 2 shows the correlation between malaria density and leucocyte differentials of infected children. After Pearson’s correlation, there was a positive but non-significant correlation between total WBC and those with mild and no malaria (Table 2). However, there was a significant negative correlation between WBC and severe malaria (p = 0.022; r = −0.712) (Table 2). There was positive but no significant association between Neutrophils and monocytes and all grades of malaria. There was a negative and significant correlation between severe malaria and lymphocyte count (p = 0.048; r = −0.647) (Table 2). There was no significant correlation between eosinophil count and mild malaria. However, there was positive and significant correlation between eosinophil with moderate (p = 0.03; r = 0.994) (Table 2) and severe malaria (p = 0.006; r = 0.825) (Table 2).

### 3.3. Ferritin and profile of oxidative biomarkers of study participants based on malaria severity

Table 3 shows the profile of oxidative stressors in children with the various grades of malaria parasitemia. The mean serum ascorbate levels of children without malaria, with mild, moderate and severe malaria was 1.23 ± 0.18 μg/dL, 0.892 ± 0.039 μg/dL, 0.534 ± 0.002 μg/dL and 0.253 ± 0.003 μg/dL, respectively. The mean serum α-tocopherol levels of children without malaria, with mild, moderate and severe malaria was 0.88 ± 0.13 μg/dL, 0.89 ± 0.29 μg/dL, 0.83 ± 0.21 and 0.79 ± 0.18 μg/dL, respectively. Using one-way ANOVA and Dunnett’s Multiple Posthoc to determine the difference between control and the three groups, there was a significant decline in serum ascorbate and within an increase in severity of malaria densities (p<0.0001). However, there was no difference in the serum tocopherol level across the 4 groups (p = 0.182) (Table 3).

The mean serum ferritin levels of children without malaria, with mild, moderate, and severe malaria was 92 ± 8.3 ng/mL, 108 ± 14.2 ng/mL, 175 ± 11.9 ng/mL and 292 ± 25.1 ng/mL. The mean serum MDA levels of children without malaria, with mild, moderate and severe malaria was 6.1 ± 0.75 nmol/mL, 7.4 ± 1.28 nmol/mL, 7.3 ± 0.99 nmol/mL and 8.2 ± 1.17 nmol/mL, respectively. Serum ferritin and MDA concentrations significantly elevated with an increase in malaria density (p<0.0001) (Table 3).

### 3.4. Cellular immune response and IL-6, IL-10 profile of study participants based on malaria severity

Table 4 shows the cellular immune status and inflammatory cytokine of the study population. The mean whole blood CD4^+^ T cell count of children without malaria, with mild, moderate and severe malaria was 710 ± 111 cells/mm^3^, 628 ± 105 cells/mm^3^, 592 ± 82 cells/mm^3^ and 390 ± 50 cells/mm^3^, respectively. The mean CD8^+^ T cell count of children without malaria, with mild, moderate and severe malaria was 428 ± 42, 325 ± 39, 282 ± 25 and 113 ± 18, respectively. Using one-way ANOVA and Dunnett’s Multiple Posthoc to determine difference between control and the three groups, there was significant decline in CD4^+^ T and CD8^+^ T cells count and within increase in severity of malaria densities (p<0.0001) (Table 4).

The mean serum IL-10 levels of children without malaria, with mild, moderate and severe malaria was 321 ± 52 pg/mL, 815 ± 105 pg/mL, 842 ± 121 pg/mL and 935 ± 115 pg/mL, respectively. The mean serum IL-6 levels of children without malaria, with mild, moderate and severe malaria was 30.3 ± 8.7 pg/mL, 83.7 ± 13.5 pg/mL, 98.5 ± 15 pg/mL and 101 ± 18.4 pg/mL, respectively. After using one-way ANOVA and Dunnett’s Multiple Posthoc to determine difference between control and the three groups, serum IL-10 and IL-6 concentrations significantly elevated with increase in malaria density (p<0.0001) (Table 4).

## 4. Discussion

Varying reports have elucidated the roles of pro-and anti-inflammatory cytokines in malaria and disease outcomes. Essentially, severe malaria has repeatedly been reported to cause decline in CD4^+^ T cell counts and induction of anemia through CD8^+^ T cell-dependent plasmodial clearance which invariably leads to rise in oxidative stress. To the best of our knowledge, this is the first comprehensive study on the T cell response, leucocyte profiles and oxidative stress level of children infected with malaria in Northern Nigeria.

Our findings revealed no significant difference in Body Mass Index (BMI) in all categories (p = 0.513), which concurred, with a research carried out among Ghanaian children and no statistically significant variation was observed in BMI (p = 0.300) [22]. This may be because neither of the studies dealt with malnourished or underweight children.

There was a positive but non-significant correlation between total White Blood Cells (WBC) and those with mild and no malaria. This is in agreement with a study carried out among Ghanaian children and found no statistical significance between those with low malaria parasitemia and those without parasitemia [22]. Likewise, it was reported in an Ethiopian study, that most of the patients with acute uncomplicated malaria usually have their mean total leucocyte count (TLC) within the normal range [23]. This may be because there was no increased total leucopoiesis but there might be increased fractional subpopulation of leucocytes as part of body immune response to malaria. However, we found a significant negative correlation between WBC among children with severe malaria (p = 0.022; r = −0.712). Eze *et al* reported no specific diagnostic indications given by the white blood cell count during malarial parasitemia [24]. In another study in Ethiopian which corroborated with our finding, it was reported that an increase in the level of parasitemia in their study was associated with a decrease in the number of leucocytes [23]. This might be due to exhaustion of white cells during malaria pathogenesis.

There was positive but no significant association between neutrophils and monocytes in all grades of malaria. This does not agree with the report of significant monocytosis (p<0.05) observed in parasitemic patients in Uganda [23] and Benin-City, Nigeria [25]. In consonance with our finding, Tatfeng and Agbonlahor reported no significant variation in neutrophils count was found in their study [25] and the study of Eze *et al* [24].

There was a negative and significant correlation between children with severe malaria and lymphocyte count. In agreement with this finding was the significantly lower lymphocyte count reported in malarial patients in the study of Muwonge *et al* [26]. In this study, no significant correlation was found between eosinophil count and mild malaria. A similar result was reported in a recent study in Benin-city, Nigeria [25]. However, there was a positive and significant correlation between eosinophil with moderate malaria parasitemia and severe malaria (p = 0.006; r = 0.825). This agrees with an earlier study by Eze and colleagues [24].

There was a significant decline in serum ascorbate with an increased in the severity of malaria densities. It is in agreement with prior study in Indian children, where a decreased serum ascorbate level was observed with an increase in malaria parasitaemia when compared with the mean ascorbate level of the healthy control group [27]. However, there was no difference in the serum tocopherol level across the 4 groups in our study (p = 0.182). In contrast to this, a report from malarial children in Uganda found serum tocopherol concentrations to be lower in children who present with acute malaria [28]. This may reflect the difference in the local diet from this study. The majority of Northern Nigerians are rural residents who are adapted to the consumption of food with low alpha-tocopherol content in milk and grains. Therefore, consumption of these may provide little or no antimalarial protection [29].

In this study, serum ferritin and Malondialdehyde (MDA) concentrations were significantly elevated with an increase in malaria density. In contrast, with the prior study in Edo state, South-south region, Nigeria, where no significant association was found between serum ferritin level and the presence of malaria parasite [30]. This may signify variations in diet as in the north-central Nigeria, where most common affordable food is starchy in nature which is deficient in iron and globulin biosynthesis. Whereas, people in Southern Nigeria have ample food stuffs that are mostly leafy that are iron rich. In contrast to this, a study in Lagos, Nigeria agrees with our findings, where they reported significantly higher serum ferritin level in malaria parasite positive subjects [31]. The findings of Nsiah *et al* on serum MDA levels in malarial children corroborated with ours. They reported a significantly higher serum MDA level in malaria parasitized children [12]. In the same study, a significant variation of MDA level was observed across control, uncomplicated and complicated malaria group Nsiah *et al* [12].

Findings from our study revealed a significant decline in CD4^+^ T and CD8^+^ T cells count as the severity of malaria parasitemia increases (p < 0.0001). This aligns with various studies on T cell response to malaria in children, where majority reported declined CD4^+^ and CD8^+^ T cell counts in malaria-infected children [32–35]. In another study in Uganda subjects, findings revealed lower CD4 T-cell counts among patients with increasing densities of malaria parasitemia [36]. This denotes a decline in CD4^+^ T cells are associated with severe malaria parasitemia.

Our study revealed serum IL-10 and IL-6 concentrations significantly elevated with increased in malaria density (p<0.0001). These agree with the study in Ugandan children, which reported significantly higher IL-6 and IL-10 only during acute malarial [37]. Similarly, significant elevated level of proinflammatory IL-6 and IL-10 was reported in Malian children with severe-malaria [38,39]. In addition, a Sudanese study reported high level of IL-10 was reported among malaria parasitemic children [40].

Proinflammatory cytokines are involved in clearance of *Plasmodium falciparum*, and very high levels of these cytokines have been implicated in the pathogenesis of severe malaria. These findings are consistent with the concept that high levels of proinflammatory cytokines, despite high levels of the anti-inflammatory cytokine IL-10, could contribute to the pathogenesis of severe malaria in children [41–46].

Malaria has been confirmed to deplete CD4^+^ T cells especially, the memory T cells. This is one of the complications encountered when mounting a successful vaccination against malaria. Malaria is also known to expand CD8^+^ T cells and T_reg_ cells and is implicated in severe malaria such as cerebral malaria because; T cell senescence and depletion markers are associated with CD8^+^ T cells. These together with the significant changes in the cytokine levels (IL-6 and IL-10) point to the debilitating effect of malaria on the immune system. This confirms that malaria leads to major immune dysfunction and affects how the body deals with subsequent malaria re-infection.

## 5. Conclusion

Based on these findings, severe malaria was significantly associated with declined CD4^+^ and CD8^+^ T cell counts, upregulation of IL-6 and high serum levels of oxidative stress biomarkers. Taken together, Ferritin, IL-6 and IL-10 could play significant roles in the inflammatory processes, severe anemia and pathologies associated with malaria.

It is recommended that future studies should investigate the direct impacts of vitamin supplements on proinflammation, other cytokines/chemokines and cellular immune function during pediatric malaria. In addition, there is a need to investigate how immunity to malaria is lost despite repeated malaria re-infections in Nigerian Children.

## Figures and Tables

**Table 1:** Characteristics of Children with various Grades of Malaria Parasitemia.

Characteristics	Mild (n = 45)	Moderate (n = 40)	Severe (n = 40)	Control (n = 40)	F value	p value
Age (Mean ± SD) Years	8.5 ± 1.9	9.2 ± 2.3	9.8 ± 2.2^c^	8.5 ± 1.5	4.05	0.008^d^
BMI (Mean ± SD) Kg/mm^2^	19.6 ± 3.5	18.8 ± 3.3^b^	19.9 ± 2.1	19.3 ± 4.3	0.77	0.513
Sex (Male: Female Ratio)	1.5:1	1:1	1:1	1:1	NA	NA
Malaria parasite density	2754 ± 125^a^	16,254 ± 2520^b^	227,651 ± 14,533^c^	NA	91.2	<0.0001^d^

α Determined by one-way ANOVA.

Key:

BMI = Body Mass Index.

a, b, c = location of significant mean difference.

a Between mild and control.

b Between moderate and control.

c Between severe and control.

d Significant difference between the control and other groups; Dunnett’s Multiple Posttest determined difference between control and specific groups.

**Table 2:** Correlation between Malaria density and Leucocyte differentials of P. falciparum infected Children.

Parameter^a^	Control (n = 40)		Mild (n = 45)		Moderate (n = 40)		Severe (n = 40)	
			
	r	p-value	r	p-value	r	p-value	r	P value
Total WBC (x10^9^/L)	0.043	0.686	0.439	0.063	−0.596	0.070	−0.712	0.022*
Neutrophils (%)	0.005	0.831	0.169	0.063	0.205	0.753	0.095	0.561
Lymphocytes (%)	0.062	0.872	0.030	0.837	−0.273	0.055	−0.647	0.048*
Eosinophils (%)	0.159	0.761	0.344	0.077	0.994	0.032*	0.825	0.006*
Monocytes (%)	0.163	0.257	0.058	0.691	0.046	0.750	0.267	0.061

Observation: Malaria density, whether mild, moderate, or severe correlated significantly with WBC, Lymphocytes and Eosinophils.

a Pearson’s correlation (*r*).

**Table 3:** Profile of Oxidative stressors in Children with various Grades of Malaria Parasitemia.

Parameter	Mild (n = 45)	Moderate (n = 40)	Severe (n = 40)	Control (n = 40)	F value	p value
			
	Mean ± SD	Mean ± SD	Mean ± SD	Mean ± SD		
Ascorbate (μg/dL)	0.892 ± 0.039^a^	0.534 ± 0.002^b^	0.253 ± 0.003^c^	1.23 ± 0.18	857.3	<0.0001^d^
Ferritin (ng/mL)	108 ± 14.2^a^	175 ± 11.9^b^	292 ± 25.1^c^	92 ± 8.3	1277	<0.0001^d^
α-Tocopherol (μg/dL)	0.89 ± 0.29	0.83 ± 0.21	0.79 ± 0.18	0.88 ± 0.13	1.64	0.182
MDA (nmol/mL)	7.4 ± 1.28^a^	7.3 ± 0.99^b^	8.2 ± 1.17^c^	6.1 ± 0.75	26.85	<0.0001^d^

Key:

MDA = Malondialdehyde.

α Determined by one-way ANOVA.

a, b, c = location of significant mean difference.

a Between mild and control.

b Between moderate and control.

c Between severe and control.

d Significant difference between the control and other groups; Dunnett’s Multiple Posthoc determined difference between control and specific groups.

**Table 4:** Cellular Immune status and Inflammatory Cytokine of Children with various Grades of Malaria Parasitemia.

Parameter	Mild	Moderate	Severe	Control	F value	P value
			
	Mean ± SD	Mean ± SD	Mean ± SD	Mean ± SD		
CD4^+^T Cell count (cells/mm^3^)	628 ± 105^a^	592 ± 82^b^	390 ± 50^c^	710 ± 111	90.3	<0.0001^d^
CD8^+^ T Cell Count (cells/mm^3^)	325 ± 39^a^	282 ± 25^b^	113 ± 18^c^	428 ± 42	643.8	<0.0001^d^
IL-10 (pg/mL)	815 ± 105^a^	842 ± 121^b^	935 ± 115^c^	321 ± 52	294.2	<0.0001^d^
IL-6 (pg/mL)	83.7 ± 13.5	98.5 ± 15	101 ± 18.4	30.3 ± 8.7	212.4	<0.0001^d^

α Determined by one-way ANOVA.

a, b, c = location of significant mean difference.

a Between mild and control.

b Between moderate and control.

c Between severe and control.

d Significant difference between the control and other groups; Dunnett’s Multiple Posthoc determined difference between control and specific groups.
